# Now is the Critical Time for Engineered Neuroplasticity

**DOI:** 10.1007/s13311-018-0637-0

**Published:** 2018-06-11

**Authors:** Chet T. Moritz

**Affiliations:** 1 Division of Physical Therapy, Department of Rehabilitation Medicine, University of Washington, Seattle, WA USA; 2 Department of Physiology & Biophysics, University of Washington, Seattle, WA USA; 3 Graduate Program in Neuroscience, University of Washington, Seattle, WA USA; 4 UW Institute of Neuroengineering (UWIN), University of Washington, Seattle, WA USA; 5 Washington Spinal Cord Injury Consortium, University of Washington, Seattle, WA USA; 6 Center for Sensorimotor Neural Engineering, Seattle, WA USA; 7 Department of Electrical Engineering, University of Washington , Box 356490, Seattle, WA 98195 USA

**Keywords:** Spinal cord injury·epidural stimulation·intraspinal microstimulation·transcutaneous stimulation·combinatorial therapies·stem cells.

## Abstract

Recent advances in neuroscience and devices are ushering in a new generation of medical treatments. Engineered biodevices are demonstrating the potential to create long-term changes in neural circuits, termed neuroplasticity. Thus, the approach of engineering neuroplasticity is rapidly expanding, building on recent demonstrations of improved quality of life for people with movement disorders, epilepsy, and spinal cord injury. In addition, discovering the fundamental mechanisms of engineered neuroplasticity by leveraging anatomically well-documented systems like the spinal cord is likely to provide powerful insights into solutions for other neurotraumas, such as stroke and traumatic brain injury, as well as neurodegenerative disorders, such as Alzheimer’s, Parkinson disease, and multiple sclerosis. Now is the time for advancing both the experimental neuroscience, device development, and pioneering human trials to reap the benefits of engineered neuroplasticity as a therapeutic approach for improving quality of life after spinal cord injury.

## Neural Devices are Becoming Ubiquitous

Traditional neuromodulation systems that stimulate the brain and spinal cord are already improving the lives of hundreds of thousands of people. Current systems typically apply stimulation in an open loop, continuous way and provide relief of symptoms, such as essential tremor or chronic pain only during operation.

The next generation of neural devices operate in a close-loop framework. These devices sense symptom onset and stimulate only when needed. Examples include the NeuroPace device for epilepsy treatment [1], and experimental devices to treat essential tremor and Parkinson disease via deep brain stimulation. The later devices are currently being tested to permit automatic detection of the signature of tremor from recordings on the brain surface, or even allow the user to think the device on and off using a simplified brain-machine interface [2, 3]. Similar to the open-loop devices, however, even these closed-loop devices require active stimulation in order to provide symptom relief.

An emerging approach aims to use closed-loop or activity-dependent stimulation to produce long-term changes in neural circuits after injury or disease. Producing such engineered neuroplasticity would mean that operation of the device is no longer necessary after effectively rewiring or otherwise repairing the disordered neural circuits in the brain or spinal cord. Exciting examples of this approach are rapidly emerging, and the potential for combining engineered plasticity with biological and pharmacological therapies is profound.

## Engineered Neuroplasticity

In the late 1940s, Donald Hebb described the principle by which neurons form and strengthen connections within neural circuits [4]. This concept of Hebbian plasticity can be summarized by the notion that “cells that fire together will wire together.” Although this process takes place naturally both during development of the nervous system and during subsequent learning, new research demonstrates that neural circuits can also be powerfully influenced by neural devices operating in a closed-loop, activity-dependent paradigm. Thus, the approach of engineered neuroplasticity aims to use devices to effect long-term rewiring of neural circuits that substantially outlast the application of stimulation.

Users of implanted visual prosthesis provide indirect evidence that neural devices can effect long-term changes in brain connectivity. James Weiland and colleagues coined the term “bioengineered neuroplasticity” to describe their observation of brain changes following prolonged use of the Argus II retinal prosthesis. Whereas the visual cortex of blind participants typically responds to both visual and tactile stimulation [5], use of a retinal prosthesis for 15 weeks led to visual cortex activity patterns more similar to people with normal vision [6].

**Fig. 1:**
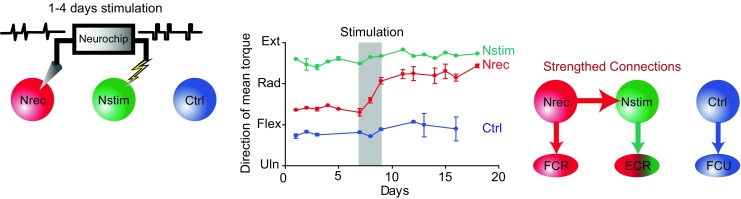
Repeated pairing of natural spiking activity with stimulation (left) leads to long-term changes in connections between the trigger neuron (red) and the stimulated site (green) that persist for 10 days (center). This is likely due to strengthening of synaptic connections between the trigger and stimulated locations (right) [7]. Reprinted by permission from Springer-Nature, long-term motor cortex plasticity induced by an electronic neural implant, Jackson et al., COPYRIGHT 2006

The first direct demonstration of engineered neuroplasticity was provided by Andy Jackson and Eberhard Fetz, who discovered that a closed-loop device could durably rewire circuits in the brain [7]. By recording the activity of one neuron, and using this activity in real-time to trigger stimulation of an adjacent brain region, they observed a robust and long-term change in neural connectivity (Fig. 1). Only 48 h of closed-loop stimulation led to changes that persisted for over 10 days. Thus, new connections in the brain were formed when two separate areas of the brain were artificially induced to fire together by the neural device, causing them to remain wired together well after the device was turned off.

The timing of closed-loop stimulation is critical to inducing long-term changes in neural circuits. Jackson and Fetz observed the greatest effect using a delay of about 20 ms between the recorded neural activity and subsequent stimulation, with less change in neural circuits using shorter and longer delays. This phenomenon of engineered neuroplasticity has since been demonstrated to improve recovery by bridging a traumatic brain injury [8].

**Fig. 2:**
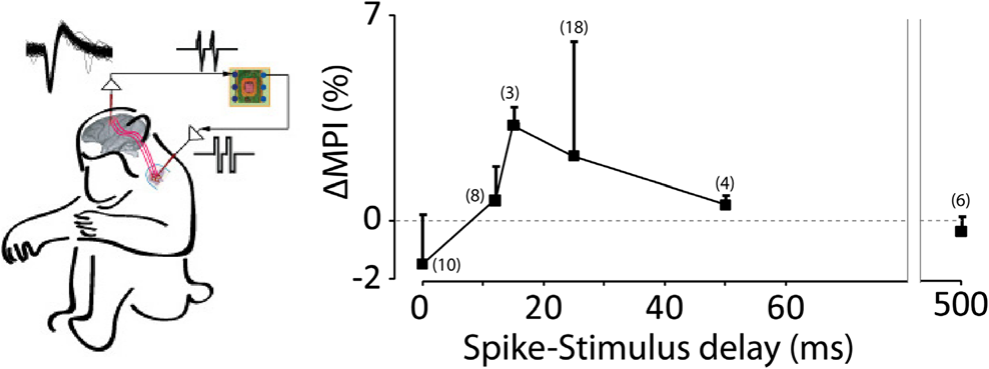
Changes in cortico-spinal connectivity via closed-loop stimulation. Spiking activity of identified cortico-motorneuronal (CM) cells are recorded from motor cortex, and used to trigger intraspinal stimulation near the target of these CM cells [9]. When the delay of the closed-loop stimulation is less than the natural conduction velocity in these circuits, connection strengths are reduced. When delays are set such that stimulation arrives shortly after the natural activity, connectivity is strengthened as measured by mean-percent increase (ΔMPI) in the spike-triggered average response of the target muscle EMG. Reprinted by permission from Elsevier-Neuron, spike-timing-dependent plasticity in primate corticospinal connections induced during free behavior, Nishimura et al., COPYRIGHT 2013

Yukio Nishimura also working with Eberhard Fetz expanded this paradigm to change the strength of connectivity between the brain and the spinal cord [9]. Identified cortico-motorneuronal cells were used to trigger intraspinal stimulation near the target of these descending projections. In this experiment, cortico-spinal connections were strengthened when the delay between the recorded brain activity and the spinal stimulation was also around 20 ms. Notably, when the device was set to zero delay, the closed-loop stimulation circuit could deliver stimulation faster than the natural ~ 6 ms conduction velocity of the cortico-motorneuronal cells. When using no delay for closed-loop stimulation, a reduction in cortico-spinal connectivity was observed (Fig. 2). This is consistent with long-term depression [10].

Thus, engineered plasticity can both strengthen and weaken natural synaptic connections, providing a robust and flexible platform for treating spinal cord injury (SCI). For example, following SCI the neural device may be set to enhance connections within the motor or somatosensory pathways. Conversely, the device may be used to reduce connectivity and excitability in aberrantly overactive pathways, such as the stretch reflex that can lead to spasticity and hyperreflexia following SCI [11]. John Wolpaw and colleagues provide an excellent example of using the timing of stimulation to modulate the strength of the spinal H-reflex in animals after SCI [12]. More recent work by the same team suggests that similar strategies are effective in human subjects after injury [13, 14].

## Neural Devices to Restore Function After Spinal Cord Injury

Neural stimulation devices can also be paired with natural activity to produce long-term recovery of function after injury [15]. Stimulation of spinal cord circuits coupled with motor retraining leads to improved function after spinal cord injury in both animal models [16–18] and human subjects [19–21]. Indeed, there is a quiet revolution in the field of spinal cord injury research, where people who were completely paralyzed are regaining the ability to stand [20], step [19, 22], and move their hands [21, 23, 24] in the presence of spinal cord electrical stimulation.

The most likely mechanism of action for both epidural and transcutaneous spinal stimulation involves activation of the dorsal root afferent fibers [25], and resulting modulation of spinal circuits. For example, spinal stimulation may bring motor circuits closer to threshold such that weak but spared descending commands can cause overt movements [23, 24]. By repeated pairing of the stimulation and movement practice over several weeks, most individuals tested to date improve their motor function during stimulation, and some participants even retain this function after the stimulation is discontinued [26]. In one case, this improvement persisted for 3 months without further treatment [24]. Further work is needed to confirm these findings in larger cohorts of individuals, such as the study of 169 individuals with cervical epidural stimulation following SCI [27]. Nonetheless, results to date provide strong evidence for engineered plasticity occurring in the cortico-spinal circuits after injury.

In addition to restoring movement during periods of spinal cord stimulation, many of these participants also had long-term gains in autonomic functions, such as bladder, bowel, thermoregulation, and cardiovascular function that are critical health problems following spinal cord injury [20, 27]. These changes in autonomic function provide some of the first evidence of device-driven engineered neuroplasticity for participants with spinal cord injury.

While the majority of studies utilize electrodes surgically implanted on the spinal cord surface, termed epidural stimulation, even stimulation applied to the skin surface over the spinal cord may lead to engineered neuroplasticity. Spasticity of the lower extremities is reduced following the application of transcutaneous electrical [28] or magnetic stimulation [29]. Most notably, after 18 sessions of skin surface electrical stimulation combined with step movement training for people with chronic spinal cord injury, stimulation was no longer required to produce the same level of volitional control as could be achieved with the stimulator active [30]. Similar results were observed for transcutaneous stimulation applied to the cervical spinal cord to improve hand function [23, 24].

Based on the improvements observed to date from non-invasive stimulation, it seems sensible to suggest that transcutaneous stimulation be tested prior to undergoing surgery to implant an epidural stimulation electrode. Transcutaneous stimulation may serve as a screening tool to see who might respond to epidural stimulation. This would be less invasive than the temporary, percutaneous leads that are placed prior to internalizing the pulse generator for treatment of chronic pain, which have been used to test the potential benefit of epidural stimulation in one case [31]. Regardless of the stimulation location selected, it is important to be vigilant about the potential for maladaptive plasticity to occur due to stimulation, such as increased spasticity or neuropathic pain.

In addition to open-loop stimulation of the spinal cord, neural technology now permits closed-loop systems capable of bridging the spinal cord injury. Neural activity can be recorded from electrodes in or near the brain to indicate the intention to move, and stimulation delivered to the paralyzed muscles to restore functional grasp in both animal models [32, 33] and human subjects [34, 35]. While direct muscle stimulation can produce fatigue, stimulation within the spinal cord results in fatigue-resistant contractions and activates muscles in functional synergies to restore both upper and lower extremity movements [36, 37]. Indeed, brain recordings can be used to trigger stimulation of the spinal cord, using a neural device to effectively bridge a lesion in the central nervous system [38, 39].

The aforementioned strategy of bridging the injury by recording in the brain and stimulating distal to the injury could be viewed largely as a prosthetic solution. Recent evidence, however, suggests that operation of such a closed-loop device can lead to long-term changes in natural connectivity bypassing the lesion. Indeed, after several years of using a brain-controlled muscle stimulator to promote hand function [35], the participant began to regain volitional control of his own hand movements without the system active (personal communication). Similar results are emerging after several years of spinal stimulation, where a participant using epidural stimulation no longer requires the device to be active to stand and control leg movements [26]. Thus, we are seeing the first evidence that delivering neural stimulation can lead to long-term recovery of movement for people with previously incurable paralysis.

The functional improvements resulting from neural devices reported to date are already life-changing for the small number of people involved in these studies. When these strategies are successfully translated to clinical practice, there will be a dramatic shift in quality of life for people with neurological disorders. For example, restoration of hand and arm function after spinal cord injury or stroke can restore independence in feeding, dressing, and grooming, thereby substantially reducing caregiver burden and costs. Restoration of bladder and bowel functions reduces life-threatening bladder and kidney infections, saves many hours each day currently devoted to bowel programs, and restores confidence and dignity, permitting engagement in social situations and travel.

## Timeline for Translation

Given that most recent breakthroughs in engineered plasticity have utilized noninvasive stimulation or implanted epidural stimulators already approved for other indications, the timeline for translation to clinical care can be unusually short. The US FDA has been very cooperative in pre-submission meetings, proactive in gathering information from the researcher community, and even approving the Expedited Access Pathway for some of these emerging technologies. For example, multisite clinical trials of transcutaneous spinal stimulation are already beginning, and assuming that safety data continue to be positive, this new treatment could be available to patients in as little as 2–3 years.

Epidural stimulators are already approved for the treatment of chronic pain. Multiple centers are currently studying the benefits of epidural stimulation for restoring movement after spinal cord injury under FDA investigational device exemption (IDE), and all are obtaining highly promising results (Louisville, UCLA, Mayo Clinic, Univ. of Minnesota, EPF-Lausanne). Therefore, efficacy need only be demonstrated for new applications of movement restoration, and perhaps also engineered neuroplasticity of autonomic function, over the next 3–5 years.

Even the development of closed-loop implantable brain stimulators are already well underway. NeuroPace recently received approval for an implant to treat epilepsy, and Medtronic is testing several low channel-count devices for treatment of essential tremor and Parkinson disease. Both startup (e.g., Neuralync, Kernel) and established companies (e.g., Galvani/GSK/Google) are ramping up to produce more complex closed-loop devices, which are expected to emerge in the next 5–10 years to enable specific and targeted engineered neuroplasticity.

It is critical to remember that even approaches targeting the peripheral nervous system (PNS) will need to consider plasticity occurring in the central nervous system (CNS) and associated ganglia. Treatments to excite or block the PNS are likely to evoke long-term changes within circuits of the CNS as homeostatic mechanisms counter the applied neuromodulation. Thus, a keen awareness of engineered neuroplasticity will likely be needed in order to produce effective neuromodulation in both peripheral and central targets to improve function in a wide range of diseases.

## Combinatorial Therapies to Enhance Plasticity and CNS Recovery

While stimulation devices can be rapidly translated to clinical practice, perhaps the greatest benefit of engineered neuroplasticity will be realized in combination with other treatments over a slightly longer time horizon. There exists a unique potential for devices to collaborate with biological and pharmacological therapies to produce targeted and robust regeneration of neural circuits.

For example, neural stem cell grafts hold great promise for restoring function to degenerating or damaged neural tissue. Approaches involve neuron cell replacement, remyelination, and environment modulation. The potential for cell replacement is demonstrated by human induced pluripotent stem cells (hiPSCs), grafted into the injured spinal cord, that extend axons long distances into the host tissue [40]. Without targeted activity, however, these grafts may not connect properly to the surrounding host tissue. Engineered devices in combination with stem-cell therapies offer the potential to create appropriate and targeted neural activity, thereby synchronizing the host and graft to promote the formation of functional connections.

Demyelination occurs after spinal cord injury [41], and during the progression of multiple sclerosis. Oligodendrocytes undergo cell death and their associated myelin sheaths degrade, which can reduce or eliminate the conduction of action potentials through long axons. This can severely limit function in many axons near a spinal cord injury site even if they are not directly damaged by the injury [42]. Although some spontaneous remyelination occurs in months following injury [43], neural activity is critical to restoring myelin sheaths and axon conduction [44]. Devices may enhance this process, such as electrical stimulation of the cortex following spinal cord injury, which leads to greater myelin protein expression via an increase in oligodendrocyte precursor cells (OPCs) and mature oligodendrocytes [45].

In addition to electrical stimulation, cell-based approaches aim to promote remyelination and restore conduction in damaged axons of the spinal cord. There are currently human trials ongoing for both the approach of using oligodendrocyte precursor cells (OPCs) [46] and Schwann cells [47, 48]. Here again, activity is critical for both inducing OPCs to remyelinate axons, and for shaping the internode distances and function of remyelinated axons [44, 49]. This reinforces the potential for neural devices to collaborate with remyelinating stem-cell therapies to fully restore function.

A final intriguing benefit of stem-cell transplantation may be to beneficially modify the host environment to induce plasticity and regeneration. This may partly explain the benefits of olfactory ensheathing cells (OECs) that secret proteins, such as metalloproteinases known to improve host axon regeneration [50]. For example, transplantation of non-neuronal cells, such as immature astrocytes may promote synaptic plasticity via similar mechanisms as newly generated tissues during development [51]. The combination of neural stimulation and plasticity-enhancing cell therapies may encourage the adult CNS to undergo productive rewiring in response to plasticity-directing stimulation from engineered devices.

Similarly, pharmacological interventions may generally enhance neuroplasticity, while engineered devices can collaborate to shape this plasticity into specific and functional circuits [52]. An example is the bacterial enzyme chondroitinase ABC (ChABC), known to dissolve perineuronal nets, thereby enhancing plasticity and even reestablishing a critical period in development of ocular dominance [53]. There is a perverse reduction in plasticity following spinal cord injury due to the accumulation of additional chondroitin sulfate proteoglycans on the perineuronal nets of synapses surrounding the injury site [54]. While ChABC alone can improve function after spinal cord injury [55–57], the combination of ChABC and an anti-body treatment to restrict the Nogo signals in myelin resulted in even greater recovery [58], demonstrating the power of synergistic therapies. In the meantime, anti-Nogo treatment alone has progressed rapidly from animal studies to human trials [59], with a phase II/III study presently underway in Europe.

It should be noted that combinatorial therapies are not always beneficial, and can even be counterproductive if administered simultaneously. For example, treatment with anti-Nogo anti-body and motor retraining must be staggered in time in order to observe benefits following stroke or spinal cord injury [60, 61]. Despite promising results, further work is needed in order to plan and optimize combinatorial therapies to engineer neuroplasticity.

An additional target for axon and neurite outgrowth is the Rho/ROCK pathway, which may benefit the damaged brain or spinal cord [62]. This pathway can be inhibited by a bacterial toxin VX-210, synthetically derived as Cethrin. Cethrin demonstrated positive results in phase I/IIa testing [63], and is currently in a phase IIb/III trial with Vertex Pharmaceuticals.

Regardless of the cell therapy or plasticity-enhancing pharmacology selected, appropriate neural activity is likely needed to create functional benefit from these studies. As examples, cord blood stem cells *combined* with locomotor retraining resulted in improved function [64]. Treatment with anti-NogoA anti-body treatment *closely followed* by treadmill training led to greater improvements than either treatment alone [60]. And preliminary results combining ChABC with spinal stimulation dramatically improved function. Therefore, the combination of neural stimulation technology and cell or molecular therapies may soon be able to leverage enhanced plasticity to drive the formation of functional connections in the damaged or degenerating central nervous system.

## Conclusion: the Spinal Cord as a Model of CNS Repair

While engineered neuroplasticity has been demonstrated to effect long-term changes throughout the central nervous system (CNS), the spinal cord after injury offers a tractable anatomical model of CNS repair. The spinal cord contains all of the cell types and neural circuits found in the brain, but arranged in a physical organization where lesions can reliably disconnects discrete spinal circuits. While studies of neuroplasticity in the brain have been largely inconclusive, the spinal cord provides an ideal testbed for understanding and optimizing plasticity-inducing treatments within the CNS. There is an opportunity to leverage advances in the fundamental understanding of engineered neuroplasticity combined with biological and pharmacological therapies to advance treatments for spinal cord injury. While directly advancing treatments for spinal cord injury, discoveries are also expected to have an impact on developing new treatment options for other neurological disorders, such as stroke, traumatic brain injury, Alzheimer’s, Parkinson disease, and multiple sclerosis. Therefore, the approach of engineered neuroplasticity may hold the key to unlocking advances in clinical treatments throughout the brain and spinal cord.
